# The recent progress of endocrine therapy-induced osteoporosis in estrogen-positive breast cancer therapy

**DOI:** 10.3389/fonc.2023.1218206

**Published:** 2023-07-07

**Authors:** Jing Xu, Bo Cao, Chunyu Li, Guohui Li

**Affiliations:** National Cancer Center/National Clinical Research Center for Cancer/Cancer Hospital, Chinese Academy of Medical Sciences and Peking Union Medical College, Beijing, China

**Keywords:** breast cancer, endocrine therapy, estrogen, osteoporosis, aromatase inhibitors

## Abstract

Breast cancer is a significant global health concern, and the discovery of endocrine therapy has played a crucial role in the treatment of estrogen-positive breast cancer. However, these therapies are often associated with osteoporosis-related adverse events, which increase the risk of fractures in breast cancer patients and can result in limited mobility and reduced quality of life. Previous studies have shown that osteoporosis is essential side effects of the breast cancer therapy, although the exact mechanisms remain mostly unclear. Current clinical treatments, such as bisphosphonates, cause side effects and may impact the therapeutic response to endocrine drugs. In this review, we explore the likelihood of endocrine therapy-induced osteoporosis in estrogen-positive breast cancer therapy and discuss the involved mechanisms as well as the therapeutic potential of drugs and drug combination strategies.

## 1 Introduction

Breast cancer is the most common cancer in women worldwide, accounting for 11.7% of total cases and 24.5% of cases in females according to the “Global Cancer Statistics 2020” (1) released by the International Agency for Research on Cancer (IARC) of the World Health Organization. It is also the leading cause of cancer deaths among women, accounting for 6.9% of total cancer deaths and 15.5% of female deaths. As of January 1, 2022, approximately 4.1 million women in the United States have a history of breast cancer (2). Current treatment options for breast cancer include surgery, radiotherapy, chemotherapy, endocrine therapy, targeted therapy, and traditional Chinese medicine (3). Surgery and radiotherapy are local treatments, and adjuvant systemic treatment is often required before or after surgery for non-metastatic breast cancer patients to reduce the recurrence rate. However, chemotherapy drugs can produce serious clinical side effects due to their high toxicity. Targeted drugs are only suitable for patients with positive human epidermal growth factor 2 (HER2). About 70% of patients with breast cancer are estrogen receptor (ER) positive and/or progesterone receptor (PR) positive. Endocrine-assisted therapy has demonstrated good efficacy in this subtype of patients and is an important treatment option for them. However, long-term use of these medications may lead to osteoporosis, joint and muscle pain (4), endometrial thickening (5), hot flusher, sweating, irritability, fatigue, insomnia and other adverse reactions. Osteoporosis increases the risk of fractures in breast cancer patients, resulting in limited mobility and reduced quality of life. The probability of osteoporosis associated with endocrine therapy is related to the type of medication used by the patient. Although lifestyle interventions and bone density assessments are the primary preventive measures, they may not always solve this problem. Medication such as bisphosphonates may be necessary if severe osteoporosis occurs, but these drugs can lead to side effects that may impact the therapeutic response to endocrine drugs. It is unclear whether osteoporosis drugs interact with endocrine drugs or otherwise impact breast cancer treatment.

Osteoporosis caused by endocrine therapy is mainly related to a decrease of estrogen in patients. Estrogen plays an essential role in the development and structure of bones, and estrogen deficiency promotes bone resorption and loss (6). Specifically, estrogen inhibits bone renewal by reducing osteoclast (OC) -mediated bone resorption and enhancing osteoblast (OB) -mediated bone formation (7). Estrogen deficiency promotes OC differentiation and bone resorption leading to bone loss, but the exact mechanism is not yet clear. This review aims to provide an overview of endocrine therapy for breast cancer, explore the occurrence of osteoporosis caused by endocrine therapy and its possible mechanism, as well as examine the prevention and treatment of this type of osteoporosis. Our objective is to promote rational drug use and improve the quality of life of breast cancer patients.

## 2 Overview of osteoporosis associated with endocrine therapy in breast cancer

Breast cancer is a malignant tumor caused by uncontrolled proliferation of ductal epithelial cells of the breast, however its specific mechanisms are not fully understood. Research has revealed that the development of breast cancer is associated with various risk factors including genetic factors and unhealthy lifestyle choices (8, 9). Breast cancer can be categorized as luminal A (ER and PR positive, HER2 negative), luminal B (ER and PR positive, HER2 positive), HER2 positive, or triple negative based on molecular pathology. Both luminal A and B breast cancers can be treated with endocrine drugs or adjuvant therapy.

Osteoporosis is a disease characterized by a decrease in bone density and an increased risk of fracture. Osteoporosis is common in menopausal women, where it is associated with bone loss due to decreased estrogen levels, and the elderly population, in which it causes unbalanced bone resorption and formation. One meta-analysis showed that the global prevalence of osteoporosis in the elderly was as high as 21.7%, with the highest prevalence in the Asian population (24.3%) (10). The analysis estimated that in 2019, the prevalence of osteoporosis in Chinese men and women aged 50+ was 6.46% and 29.13%, respectively (11). This data suggests that elderly women have a higher incidence of osteoporosis.

The strategy of endocrine therapy is to reduce the production of estrogen or the binding level of estrogen to ERs in tumor cells, thereby inhibiting the growth and proliferation of tumor. Estrogen stimulates OBs and inhibits OCs, therefore the main cause of endocrine treatment-related osteoporosis in breast cancer is bone loss induced by estrogen depletion. Patients receiving this treatment often experience adverse effects such as estrogen deficiency-related osteoporosis-induced brittle fractures. The risk of osteoporosis in patients varies based on the targets of various endocrine drugs. The most accurate clinical indicator of osteoporosis is the measurement of bone mineral density (BMD) or bone mineral content (BMC) using dual-energy X-ray absorptiometry (12). BMD can predict bone fracture risk and is typically measured at the femoral neck, total hip, or lumbar spine. T-score is generally used to evaluate osteoporosis clinically. Patients with T-score lower than 2.5 can be preliminarily diagnosed as suffering from osteoporosis.

The main anti-estrogenic drugs commonly used in clinical practice are tamoxifen (TAM), toremifene, raloxifene, and fulvestrant. These drugs compete with estrogen in the body by binding ERs. TAM, despite being an ER agonist in the bone and uterus, acts as an antagonist in breast tissue and is considered a selective estrogen modulator (SERM) due to its tissue-specific effects (7, 13). Toremifene is a chlorinated derivative of TAM and is also used in breast cancer treatment. TAM is one of the most commonly used endocrine drugs in the treatment of breast cancer and has been used for around 50 years. A 2020 nationwide retrospective cohort study conducted by Lee Jihyound and other Korean researchers using data from the Health Insurance Review and Assessment Service (HIRA) concluded that TAM does not increase the risk of osteoporosis and osteoporotic fractures in breast cancer patients under 40 years of age and has a protective effect on bones in breast cancer patients aged 40 to 49 years (14). However, another research suggested that the 5-year rate of osteoporotic fracture for patients treated with tamoxifen was 6.9% (15). There was no significant difference (HR = 1.09; 95% CI = 0.96–1.23, p-value = 0.18) from the data of aromatase inhibitors (7.5%) mentioned in the paper. So more extensive real-world studies of TAM are needed to provide sufficient evidence of its association with osteoporosis in breast cancer patients who use the drug. Fluvestrant is a selective ER down-regulator (SERD) and induces the degradation of ERs by competitively binding with them. Although no studies have been conducted to correlate the use of fulvestrant with osteoporosis, there is a risk that the drug may cause osteoporosis based on its therapeutic mechanism. Thus, BMD is typically measured during the clinical use of fulvestrant to monitor the development of osteoporosis.

Aromatase inhibitors are often the first-choice treatment in postmenopausal women with breast cancer. When combined with aromatase, these drugs work by blocking the conversion of androgens into estrogen in the body, which reduces the estrogen levels and ultimately inhibits the growth of tumor cells. However, the use of aromatase inhibitors exacerbates the age-related reduction in BMD (16) due to the link between estrogen levels and bone health (**Table 1**) (20). Anastrozole, letrozole, and exemestane are frequently used aromatase inhibitors, with the latter being a steroid. Exemestane is structurally similar to androstendione, the natural substrate of aromatase, and binds irreversibly to the aromatase to inactivate it. A randomized phase III trial showed no significant differences in efficacy or side effects among these three aromatase inhibitors (17). On the other hand, a meta-analysis revealed that all aromatase inhibitors had a higher incidence of osteoporosis compared to tamoxifen, but that the incidence of osteoporosis was lower in patients using exemestane than in those using anastrozole (OR: 0.8594, 95% CI: 0.5766-1.168) and letrozole (OR: 0.7358, 95% CI: 0.4301-1.307) (21). There may be differences between the two classes of aromatase inhibitors in terms of bone-related metabolism. Anastrozole use accelerates bone loss in patients with breast cancer, but the associated bone loss appears to be manageable and partially reversible with discontinuation of treatment, particularly in the lumbar spine (22–24). Irene E.G. van Hellemond et al. (18) concluded from a phase III DATA study that adjuvant use of anastrozole after 2-3 years of tamoxifen decreased BMD in postmenopausal breast cancer patients. In contrast, prolonged anastrozole treatment did not increase the incidence of osteoporosis. Similarly, patients treated with exemestane had a 2.6% decrease in spinal BMD from baseline at 6 months and only a 0.2% decrease from 6 months to 12 months (25).

**Table 1:** Incidence of osteoporosis associated with aromatase inhibitors.

Trial(Ref)	Follow-up time	Enrolled patients	Design	Osteopenia	Osteoporosis
NCT00541086 (17)	60 months	3697	Anastrozole		21%
			Exemestane		22%
			Letrozole		22%
NCT00301457 (18)	7 years	1860	6 years anastrozole after 2 to 3 years of tamoxifen	46.9%	9.5%
			3 years anastrozole after 2 to 3 years of tamoxifen	42.8%	9.1%
NCIC CTG MA.27 (19)	4.1 years	7576	Exemestane		31%
			Anastrozole		35%

LHRH analogues, such as goserelin, leuprorelin and triptorelin, are commonly used ovarian castrating drugs. This class of drug inhibits the secretion of luteinizing hormone and follicle-stimulating hormone in the anterior pituitary gland, resulting in significantly reduced estrogen in patients. Thus, these drugs are most suitable for premenopausal women. However, the use of these drugs can also results in decreased BMD, leading to osteopenia or osteoporosis (26). While these drugs have achieved some therapeutic success, their use is limited and there is a need for development of new breast cancer treatments.

Recently, CDK4/6 inhibitors have entered the clinic and have shown promise in treating ER-positive and HER2-negative advanced breast cancers. CDK4/6 inhibitors are cyclin-dependent kinase inhibitors that prevent tumor cells from entering the S phase from the G1 phase by acting on the cyclin-CDK4/6 complex, thereby inhibiting tumor growth. Palbociclib, ribociclib, abemaciclib, and trilaciclib are currently available CDK4/6 inhibitors. To date, there have been no reported cases of osteopenia or osteoporosis in breast cancer patients caused by CDK4/6 inhibitor use, although more research is required.

## 3 Mechanisms of endocrine drugs in breast cancer and drug-related osteoporosis

Several drugs have been studied that target ERs, which are activated by the female sex hormone estrogen. ERs are divided into two receptor subtypes, ERα and ERβ, which differ primarily in location (27). ERα is mainly found in breast cells and bone (28), making it a central target in the pathogenesis of breast cancer (29). There are two main estrogen synthesis pathways in humans: direct synthesis and secretion by the ovaries and synthesis from aromatase within the adrenal gland, fat, and other tissues. The former pathway is most prevalent in premenopausal women, while the latter is most common in postmenopausal women. Furthermore, there are three main types of estrogen in women: estrone (E1), estradiol (E2 or 17β-estradiol), and estradiol (E3). E2 is the most potent estrogen and is the main product of female premenopausal biosynthetic reactions (27).

PRs are typically co-expressed with ERs, but their role in the development of breast cancer is not fully understood. PR antagonists have not been clinically used due to serious side effects (30). The combination of estrogen and progesterone significantly affects metabolism, with estrogen tending to target tumor-promoting genes that alter glucose metabolism and progesterone targeting fat storage (31). Postmenopausal women who use estrogen and progesterone together have an increased risk of breast cancer (26%) (32) and increased breast cancer mortality (33). Additionally, a case cohort study of postmenopausal women demonstrated an increased risk of breast cancer with elevated circulating progesterone levels (16%) (34). Thus, PR plays an important role in the occurrence and progression of breast cancer. PR has two subtypes, PRA and PRB, and studies have found that a PR can bind to an ER after it is combined with its agonist, thus affecting ER-related behaviors (35). Both natural and synthetic progesterone antagonize the mitotic effect of estrogen (36). PRB is activated by a ligand, and it then activates the transcription factor EB which induces autophagy in breast cancer cells (37). Moreover, PRs can interact with STAT1 to inhibit IFN-induced STAT1 phosphorylation, thereby inhibiting carcinoma development (38). Some evidence suggests that the role of progesterone in breast tumor promotion and growth may be mediated by a receptor activator of NF-κB ligand (RANKL)-dependent effect. RANKL inhibition does not directly interfere with progesterone and PR interaction (39).

**Figure 1:**
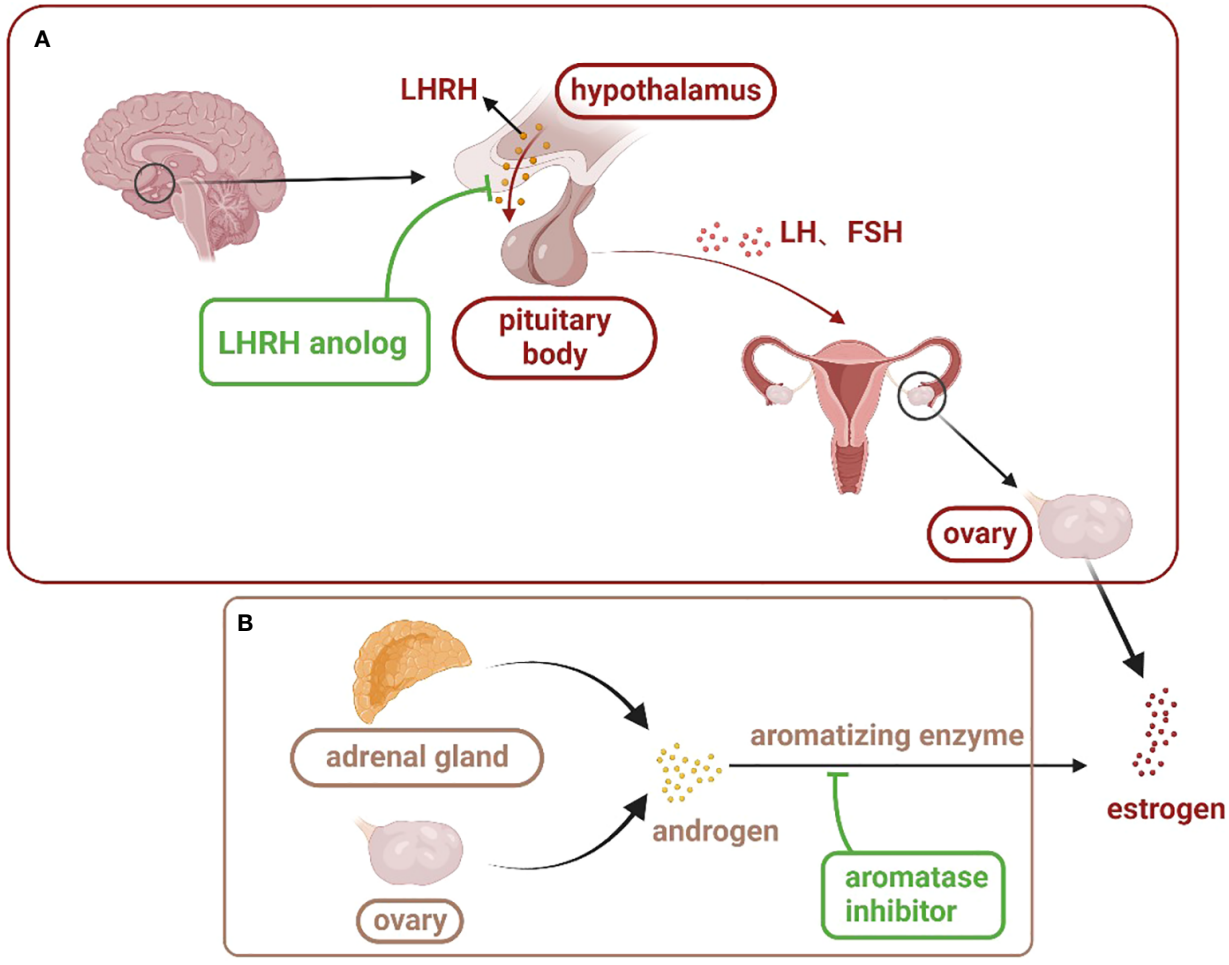
Estrogen production pathway in women: **(A)** is the main estrogen production pathway in premenopausal women. LHRH analogues inhibit the action of LHRH on the pituitary gland through competitive binding, thus inhibiting the secretion of estrogen in the ovary. **(B)** is the main estrogen production pathway in postmenopausal women. Aromatase inhibitors bind to androgens and prevent their conversion to estrogen. (LHRH, luteinizing hormone releasing hormone; LH, luteinizing hormone; FSH, follicle-stimulating hormone).

**Figure 2:**
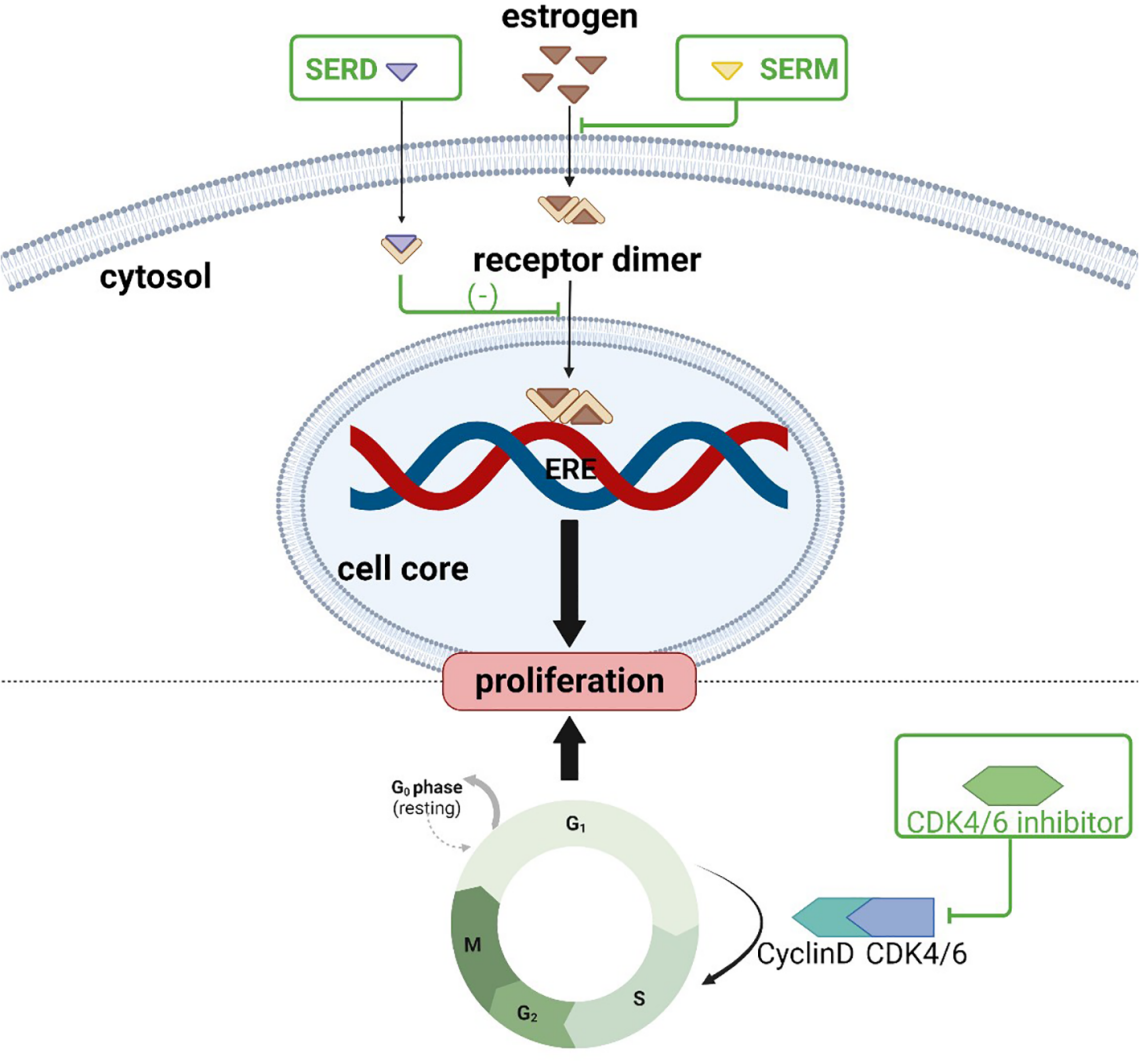
Action sites of anti-estrogen and CDK4/6 inhibitors: SERM and SERD bind to estrogen receptors, inhibiting downstream pathways and preventing tumor cell proliferation; CDK4/6 inhibitors prevent dividing tumor cells from the G1 phase into the S phase.

Endocrine therapies for breast cancer can be divided into several categories based on the production pathway and role of the hormone receptors in the occurrence and progression of breast cancer (**Table 2**), including antiestrogen drugs (selective ER regulator, selective ER downregulation), aromatase inhibitors, luteinizing hormone releasing hormone analogues (LHRH analogues), progesterone drugs, and CDK4/6 inhibitors. The action and targets of these drugs are shown in **Figures 1**, **2** (produced by BioRender, and the website is https://www.biorender.com/).

**Table 2:** Commonly used drugs and targets for endocrine therapy of breast cancer.

Type	Drugs	Target
Anti-estrogens	Tamoxifen, toremifene, raloxifene, fulvestrant	ER
Aromatase inhibitor	Anastrozole, letrozole, exemestane	Aromatase enzymes
LHRH analogues	Goserelin, leuprorelin, triptorelin	LHRH receptor
Progestogens	Megestrol	PR
CDK4/6 inhibitor	Palbociclib, ribociclib, abemaciclib, trilaciclib	CDK4、CDK6

**Figure 3:**
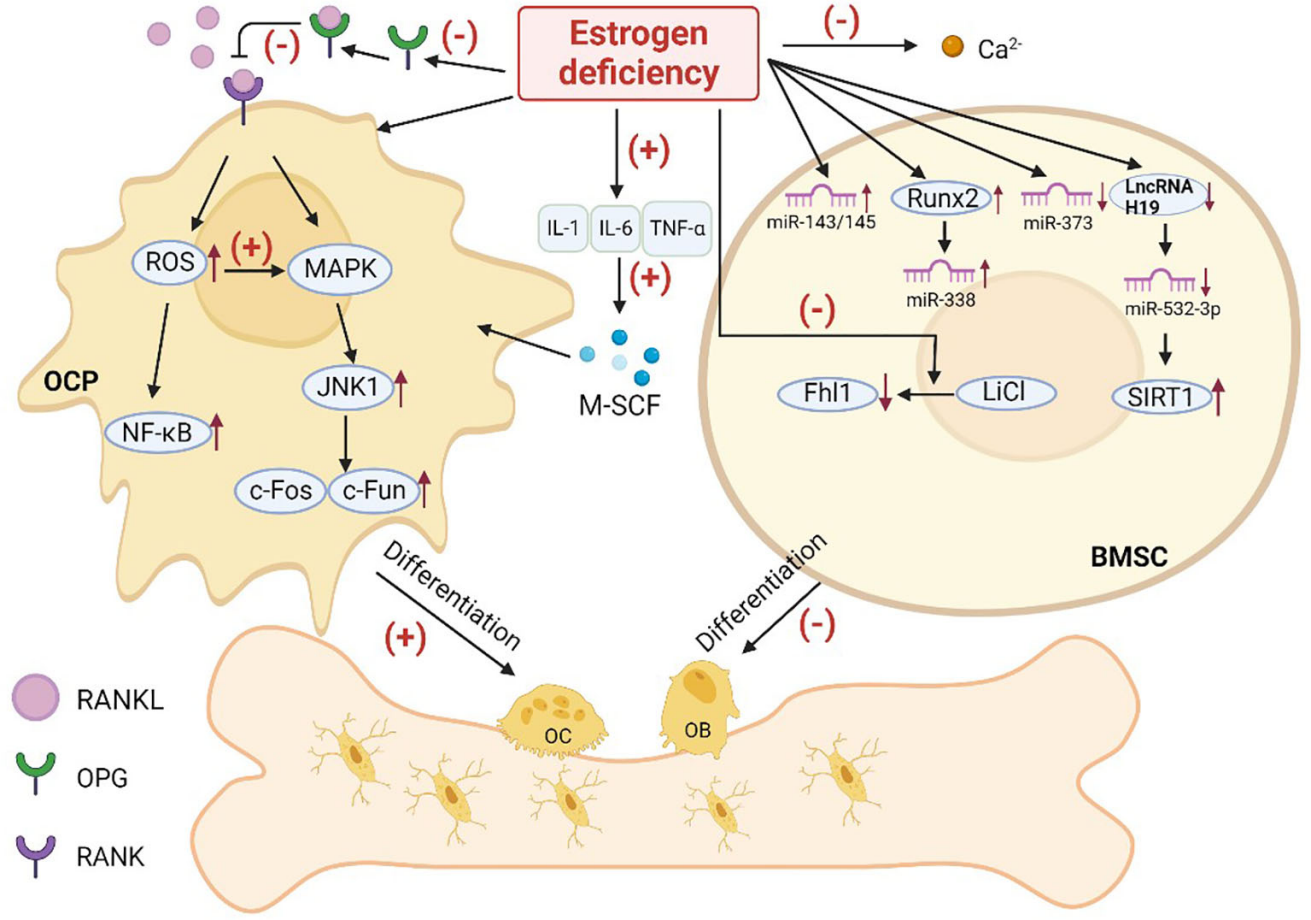
Possible pathways of osteoporosis caused by estrogen deficiency. (OC, osteoblast; OB, osteoblast; OCP, osteoclast precursor; BMSC, bone marrow mesenchymal stem cell; OPG, osteoprotegerin; RANKL, receptor activator of NF-κB ligand; RANK, receptor activator of NF-κB; IL-1, interleukin-1; IL-6, interleukin-6; TNF-α, tumor necrosis factor-α; M-SCF:; ROS, reactive oxygen species).

Estrogen is an important hormone that helps regulate the metabolic process of bone by inhibiting OCs thereby preventing osteoporosis. Bone loss caused by endocrine therapy in patients with hormone receptor positive breast cancer is mainly due to estrogen deprivation (40). At the osteocyte level, estrogen inhibits OC differentiation which reduces the number of active remodeling units. The mechanism of osteoporosis associated with endocrine therapy in breast cancer is related to changes in cytokines, the RANK pathway, Wnt pathway, and MicroRNA after estrogen level reduction (**Figure 3**). However, the mechanism of its action in the skeleton caused by crosstalk between various pathways in skeletal cells has not been fully elucidated.

### 3.1 Cytokine

Previous studies have suggested that estrogen may exert bone-protective effects by inhibiting inflammatory cytokines, such as interleukin-1 (IL-1), IL-6, and tumor necrosis factor-α (TNF-α), that promote OC formation and bone resorption. Estrogen selectively regulates IL-1R isoforms in OC or OC-like cells thereby decreasing their IL-1 responsiveness and survival (41). Conversely, this IL-1 inhibition may be lost as estrogen levels decline, resulting in OC-mediated bone loss. Estrogen deficiency leads to increased secretion of IL-1 and TNF, increased macrophage colony-stimulating factor (M-CSF) (42), and increased osteoclasts. Animal studies have shown that IL-6-deficient ovariectomized (OVX) female mice do not experience significant changes in bone mass and are protected from bone loss caused by estrogen depletion (43).

### 3.2 RANK/RANKL/OPG pathway

Prior research has shown that the effect of estrogen on bone is related to the RANKL pathway, which promotes the transformation of osteoclast precursors (OCPs) into mature OCs. Activators of RANKL signaling include reactive oxygen species (ROS), the NF-κB pathway, and mitogen activated protein kinase (MAPK) (44).

The protein osteoprotegerin (OPG) can bind to RANKL within the bone remodeling environment to prevent excessive OC formation. Estrogen activates p38α (a p38MAPKs) to maintain OPG gene expression and production in bone marrow mesenchymal stem cells (BMSCs) to protect the bone (45, 46).

Reactive oxygen species (ROS), including superoxide ion (O^2−^) and hydrogen peroxide (H_2_O_2_), are important components that regulate OC differentiation. The downstream target of ROS remains unclear, but increased oxidative stress may promote the cellular activity of OCs by triggering the NF-κB and MAPK signaling pathways (47). Under normal physiological conditions, ROS produced by OCs stimulate and promote bone tissue absorption (48). However, deficiency of the potent antioxidant estrogen leads to the accumulation of ROS in the body which in turn affects osteogenic differentiation of stem cells and the formation of osteoporosis (49).

Estrogen can inhibit RANKL expression in bone lining cells (50), and the reduced rate of RANKL formation subsequently reduces the differentiation of OCPs into OCs. Another mechanism by which E2 downregulates OC formation is by decreasing OCP reactivity to RANKL (51). E2 down-regulates the activation of Jun N-terminal kinase1 (JNK1). This decreased JNK1 activity leads to decreased nuclear levels and DNA binding of the key OC transcription factors c-Fos and c-Jun, which weakens the differentiation ability of OCPs. It was shown that when estrogen levels were reduced *in vivo*, RANKL expression was increased, the response of OCPs to free RANKL was enhanced, and bone resorption was increased.

E2 inhibits OC differentiation and stimulates mature apoptosis (52) by increasing the expression of transient receptor potential vanilloid 5 (TRPV5) channels (53). The specific mechanism is as follows: E2 enhances the expression of TRPV5 through the interaction of ERα with NF-κB, and NF-κB can directly bind to the TRPV5 promoter region from −286 nt ~−277 nt fragments. When estrogen is deficient, the expression of the TRPV5 channel weakens, OC differentiation enhances, and the risk of osteoporosis increases.

### 3.3 Wnt pathway

The Wnt pathway is highly conserved and present in both invertebrates and vertebrates. It plays a critical role in various physiological processes during early animal embryo development, including organ formation and tissue regeneration. The Wnt signaling pathway also regulates proliferation and differentiation of OBs by influencing gene transcription. Estrogen deficiency reduces GSK3β phosphorylation in OBs, leading to inhibition of the Wnt/β-catenin pathway and decreased OB proliferation (54). The Wnt agonist LiCl has been shown to induce the expression of the Fhl1 gene, which in turn promotes the expression of osteogenic markers such as Runt-associated transcription factor 2 (Runx2), osteocalcin (OCN), and osteopontin (OPN). This ultimately enhances OB differentiation significantly. Estrogen cannot directly act on OBs, but it can coordinate with LiCl to stimulate Fhl1 expression and promote OB differentiation (55).

### 3.4 MicroRNA

MicroRNA (miRNA) is a type of non-coding single-stranded RNA that plays a crucial role in the regulation of post-transcriptional gene expression and is essential for mammal bone development (56). Overexpression of miR-373 can promote the differentiation of BMSCs into OBs and reverse the decreased osteogenic differentiation ability of BMSCs caused by osteoporosis. Estrogen deficiency causes decreased miR-373 expression (57), significant enrichment of the miR-338 cluster (58), and elevated expression of miR-143/145 (59), leading to a decreased differentiation of BMSCs into OBs. Furthermore, estrogen can decrease the expression of miR-532-3p through up-regulation of long noncoding RNA H19 (lncRNA H19). LncRNA H19-mediated disruption of the miR-532-3p/SIRT1 axis has been shown to induce osteogenic differentiation of BMSCs which alleviated osteoporosis in OVX rats (60). However, osteogenic differentiation induced by this pathway is reduced if estrogen is deficient *in vivo*. Estrogen up-regulates miR-27a, which inhibits peroxisome proliferator-activated receptor gamma (PPARγ) in OCs and adenomatous polyposis coli (APC) expression, resulting in decreased OC production and reduced bone resorption (61). The estrogen-activated ERα pathway inhibits the production of miR-21 (62), which enhances Fasl protein to stimulate caspase-3 activity and induces the Fas/FasL system to regulate the lifespan of mature OCs (63), thereby inducing OC apoptosis. However, studies have also shown that estrogen deficiency-related decreased expression of miR-128 can lead to decreased OC production (64), thereby delaying or preventing the occurrence of osteoporosis. Overall, estrogen can inhibit the activity of OCs, reduce bone resorption, promote OB formation, and enhance bone formation by interacting with various miRNAs in OBs and OCs. Further research is required to uncover the regulatory pathways of estrogen and miRNAs on bone remodeling in various bone cell types.

In addition, estrogen directly affects calcium absorption in the gut, and calcium plays an important role in bones (65). Therefore, low estrogen levels can lead to insufficient calcium intake in the body, which is a primary cause of osteoporosis.

These targets and pathways associated with osteoporosis are also present in other organs and tissues. Therefore, the design of drugs for the treatment of osteoporosis for these targets should also consider the side effects on organs and tissues. Specific targets of bone metabolism are necessary to explore for better treatment of bone-related diseases.

## 4 Strategies to prevent or treat osteoporosis caused by endocrine therapy for breast cancer

Although osteoporosis cannot be cured, it can be prevented, slowed down, or stopped through various means, including increasing bone formation and inhibiting bone resorption. Biochemical markers of bone turnover can indicate the events that occur during the bone remodeling cycle and are divided into bone formation and bone resorption markers (66). Clinical practices currently use serum osteocalcin, serum bone alkaline phosphatase (BAP), urinary N-terminal peptide of type 1 collagen (NTx), and urinary C-terminal peptide of type 1 collagen (CTx) to monitor bone turnover. Various guidelines (67–69) have recommended reducing the risk of fragility fractures through screening, life interventions, and pharmacological treatment as a primary goal of osteoporosis prevention and treatment. For breast cancer patients on endocrine medications, especially aromatase inhibitors, regular monitoring of BMD is paramount. Appropriate medications should be used to prevent the development of osteoporosis, and the patients who have been diagnosed with osteoporosis require medications to stop continued bone loss and prevent fractures. Anti-resorptive agents and anabolic agents are common medications used to prevent and treat osteoporosis (**Table 3**). Importantly, osteoporosis associated with breast cancer endocrine therapy differs from conventional osteoporosis management due to the need to balance the outcome and course of the cancer treatment. However, due to limited information on osteoporosis management in cancer patients, current clinical strategies for endocrine treatment-related osteoporosis in breast cancer reference normal osteoporosis (75).

**Table 3:** Osteoporosis treatment drugs and targets.

Type	Category	Drug	Target spot
Antiresorptive agent	Bisphosphonates	Alendronate Ibandranate Risedronate Zoledronate	hydroxyapatite
	RANKL inhibitor	Denosumab	RANKL
	Calcitonin	Calcitonin	OB
	Hormone	Estrogen	ER
	SERMs	Raloxifene	ER
	Tissue-specific estrogen complex	Bazedoxifene	ER
Anabolic agent	Sclerostin inhibitor	Romosozumab-aqqg	Sclerostin
	Parathyroid hormone analogs	Teriparatide	OC
	Parathyroid hormone-associated protein analogues	Abaloparatide	OC
Potential drug	Active substance	Sirt3 inhibitor (70)	Sirt3
	Natural compound	Isosinensetin (71)	ROS
		Obacunone (44)	MIF
	Active ingredients of traditional Chinese medicine	Boldine (72)	ATF pathway
		Icariside I (73)	OC, OB
		Corylifol A (74)	ROS

### 4.1 Lifestyle intervention

In order to prevent or treat osteoporosis, breast cancer patients should quit using tobacco products and alcohol (76), take adequate calcium and vitamin D supplements (77), and exercise moderately. It may also be beneficial for patients to consume appropriate antioxidant functional foods. For example, one study reported that green tea consumption and exercise were negatively associated with osteoporosis (78). Additional studies have found that tea consumption reduces the risk of osteoporosis (79), and that weight-bearing and resistance exercise maintains BMD and improves the quality of life in postmenopausal women with low bone mass (80). However, there was not a strong BMD improvement in breast cancer patients.

### 4.2 Antiresorptive agent

Antiresorptive agents, including bisphosphonates, RANKL inhibitors, estrogens and SERMs, are the preferred drugs for the treatment of osteoporosis (81). **Table 4** provides a list of clinical trials that have evaluated the use of antiresorptive agents. A recently published real-world study showed that early postmenopausal women with ER-positive breast cancer who were treated with aromatase inhibitors in combination with anti-bone resorptive therapy experienced a significant increase in both femur and lumbar BMD after 24 months (6.28%, 7.79%, respectively) (85). Anti-bone resorption therapy can significantly improve BMD in postmenopausal women with early breast cancer who are using aromatase inhibitors, thereby preventing bone loss. One study found that the combination of letrozole and zoledronic acid inhibited the growth of and induced apoptosis in MCF-7 and T-47D human breast cancer cell lines (86). As a bisphosphate, zoledronic acid can be used in the treatment and prevention of osteoporosis, though the clinical effect of the combined use of letrozole and zoledronic acid requires additional studies. A 24-month randomized controlled trial showed increased BMD and decreased bone resorption in total hip joint (+1.81%) and spine segments 1 to 4 (+2.85%) in subjects taking risedronate, calcium, and vitamin D without exercise (87). This indicates that bisphosphonates, calcium, and vitamin D are effective treatments for bone loss in postmenopausal breast cancer patients with hip and spine bone loss. However, oral bisphosphonates can have side effects that may cause patients to discontinue the drug midway through its use. For example, bisphosphates can cause irritation to the mucosa of the gastrointestinal tract, leading to esophagitis, dysphagia, and gastric ulcers (75). Additionally, some patients have experienced hypocalcemia (88). One study reported that approximately 20% of patients who received intravenous zoledronic acid experienced side effects including flu-like syndrome (69%), arthralgia (7.7%), and even renal failure (89).

**Table 4:** Clinical trials and efficacy of antiresorptive agents.

Trail(Ref)	Period	Enrolled patients	Design	BMD change		
				Total hip	Lumbar spine	Femoral neck
NCT02616744 (82)	2 years	171	Placebo 150mg monthly	-0.19(-10.8%)	-0.09(-5.1%)	
			Ibandranate 150g monthly	+0.09(+5.3%)	+0.28(+16.6%)	
Prospective randomized observational study (83)	24 months	84	Anastrozole		-4.8%	-3.5%
			Anastrozole plus risedronate		+6.86%	+2.8%
NCT00485953 (84)	24 months	109	Oral risedronate 35 mg once weekly	+0.6	+2.3	+0.4
			Oral placebo 35 mg once weekly	-2.7	-1.7	-2.1

Denosumab specifically targets RANKL, inhibits OC activation and progression caused by related pathways, reduces bone resorption, and increases BMD in the treatment of postmenopausal women with osteoporosis. The use of denosumab has been reported to increase BMD in the lumbar spine, hip, and femoral neck in breast cancer patients treated with aromatase inhibitors (90). Results from a randomized controlled trial exhibited that subcutaneous administration of denosumab every 6 months significantly reduced clinical fracture rates and significantly delayed time to first fracture in postmenopausal patients with hormone receptor-positive early-stage breast cancer treated with adjuvant aromatase inhibitors (91). However, denosumab does not specifically target bone (92), therefore it inhibits the RANKL pathway in other tissues. Its common side effects include hypocalcemia (93), nasopharyngitis, upper respiratory tract infections, urinary tract infections, arthralgia, headache, constipation, and skin rash (75).

Raloxifene, a selective hormone receptor modulator (SERM), is widely used for the prevention and treatment of postmenopausal osteoporosis (94). Results of a randomized clinical trial showed that 3 years of raloxifene treatment maintained BMD and reduced bone turnover. Moreover, raloxifene increased BMD in the spine and femoral neck and reduced the risk of vertebral fractures in postmenopausal women with osteoporosis (95). An animal trial also demonstrated that the osteoporosis drug lasoxifene has the potential to treat ER-positive patients who are resistant to aromatase inhibitors (96).

Hormone therapy is not a feasible treatment option for osteoporosis associated with endocrine therapy in breast cancer patients, as estrogen can promote the progression of breast tumors and affect the treatment of breast cancer.

### 4.3 Anabolic agent

Antiresorptive agents do not restore the bone mass and structure that is lost due to increased bone remodeling. On the other hand, anabolic agents increase the likelihood of new bone formation (97). In a randomized controlled trial, the most commonly used parathyroid hormone analog, Teriparatide, was found to be associated with a lower risk of new vertebral fractures (5.4%) at 24 months of use compared to risedronate (12.0%) in postmenopausal women with severe osteoporosis (98). However, some scientists argue that comparison between these two drugs is not clinically significant as they are administered in different ways (99). A recent meta-analysis revealed that teriparatide was linked to a reduced risk of vertebral fracture compared to bisphosphonate (RR= 0.57, 95% CI: 0.35-0.93, P = 0.024), and that the drug increased the mean percentage change in femoral neck BMD at 18 months (P < 0.05) (89). In conclusion, teriparatide is an effective drug for reducing the risk of vertebral fractures in postmenopausal women with osteoporosis, and it can increase the BMD of lumbar spine and femoral neck in the long term.

### 4.4 Potential drug development

Research on the mechanisms of osteoporosis caused by estrogen deficiency *in vivo* may lead to new breakthroughs in the development of osteoporosis drugs. Studies have shown that Connexin 43 half-channels (Cx43 HCs) demonstrate protective effects on bone cells and can inhibit bone loss in estrogen-deficient mice following ovariectomy (100). Another promising avenue of research is the mitochondrial protein deacetylase, Sirtuin-3 (Sirt3), which has a high mitochondrial content in OCs. Inhibition of this enzyme can damage mitochondria-related functions in OCs and reverse the increased bone resorption and bone mass loss caused by estrogen deficiency (70). Sirt3 inhibitors are promising for osteopenia caused by endocrine therapy in breast cancer.

Scientists are also investigating natural compounds and active ingredients in traditional Chinese medicine as potential drugs to treat osteoporosis. For instance, isosinensetin, a flavonoid present in citrus fruits with antioxidant properties, has been shown to reduce bone loss in OVX mice and alleviate estrogen deficiency-induced osteoporosis in mice (71). Obacunone, a small molecule with a wide range of biological activities, can inhibit the formation and absorption function of OCs *in vitro* by targeting inhibitory factor of macrophage migration inhibitory factor (MIF) (44). Thus, it is expected to be an effective drug for relieving osteoporosis caused by estrogen deficiency. Traditional Chinese medicine also shows promise in treating osteoporosis by invigorating the kidneys and spleen. One animal study showed that Bushen Jianpi Decoction improved bone loss without affecting estrogen levels in mice and may increase the sensitivity of breast tumor cells to endocrine drugs, thereby improving efficacy (101). Boldine, an alkaloid isolated from the Bordeaux tree, has a protective effect against bone loss in mice caused by estrogen deficiency (72) by inhibiting OC formation induced by activator of nuclear factor κB ligand receptor by impacting the AKT signaling pathway. Icariside I (GH01), a novel isopentenyl flavonoid isolated from epimedii, was shown to effectively ameliorate estrogen deficiency-induced osteoporosis and strengthen the trabecular and cortical bone in OVX mouse models by simultaneously regulating OB and OC differentiation (73). Eucommia ulmoides and psoralea were commonly used drugs (102). Corylifol A, an isoflavone isolated from psoralea fruit, inhibits OCs and absorption by inhibiting intracellular ROS levels to prevent bone loss caused by estrogen deficiency (74). Psoralen extracted from psoralen seed has been studied for its in anti-tumor effects, but it has also been shown to impact certain pathways that may result in anti-osteoporosis effects (103). Acid polysaccharide EuOCP3 extracted from Eucommia ulmoides skin can restore cortical bone thickness, increase mineralized bone area, increase the number of OBs, and reduce the number of OCs on the cortical bone surface in osteoporotic mice (104). The total flavones of Radix osteoblastum are widely used in the treatment of postmenopausal osteoporosis (105) are expected to emerge as a new treatment for breast cancer-related osteoporosis. There are also many effective Chinese medicine ingredients for osteoporosis, such as resveratrol, puerarin, astragaloside IV, and Danshensu (106).

## 5 Summery and prospects

Endocrine therapy for breast cancer can lead to osteoporosis, particularly in postmenopausal women who use aromatase inhibitors, which impacts patient quality of life. There are limited data on the management and prevention of cancer-related osteoporosis, with no drugs specifically targeted for these patients. More clinical studies are needed to provide more effective and safe treatment options. Traditional Chinese medicine has great potential in the prevention and treatment of osteoporosis caused by endocrine therapy in breast cancer. As the acquired and intrinsic resistance mechanisms of endocrine therapy drug and CDK4/6 inhibitors is gradually revealed, there is a need to develop novel endocrine drugs with higher efficacy and fewer side effects for breast cancer patients (107). Blood vessels and lymphatic vessels are important components of bone. Some studies have shown that the vascular system plays an important role in the process of bone metastasis (108). It is necessary to explore their relationship with osteoporosis to provide new treatment strategies. An animal study showed that Substance P(SP), an endogenous neuropeptide, blocked H-type vascular loss and sustained angiogenic factor enrichment in pretreated OVX mice. SP can mediate early vascular protection and inhibit bone density loss (109). The relationship between bone lymphatics and osteoporosis remains to be further explored. Additionally, the pathogenesis of breast cancer is not yet fully understood, and there is a need for more effective therapeutic drugs to reduce the occurrence of adverse reactions related to endocrine therapy.

## Author contributions

JX collected the related paper, drafted, and revised the manuscript. BC participated in the design of the review, and helped to draft and revise the manuscript. CL and GL designed the review. All authors contributed to the article and approved the submitted version.
